# Trends in Total Binge Drinks per Adult Who Reported Binge Drinking — United States, 2011–2017

**DOI:** 10.15585/mmwr.mm6902a2

**Published:** 2020-01-17

**Authors:** Dafna Kanny, Timothy S. Naimi, Yong Liu, Robert D. Brewer

**Affiliations:** ^1^Division of Population Health, National Center for Chronic Disease Prevention and Health Promotion, CDC; ^2^Section of General Internal Medicine, Boston Medical Center, Massachusetts.

Each year, excessive drinking accounts for one in 10 deaths among U.S. adults aged 20–64 years (*1*), and approximately 90% of adults who report excessive drinking* binge drink (i.e., consume five or more drinks for men or four or more drinks for women on a single occasion) (*2*). In 2015, 17.1% of U.S. adults aged ≥18 years reported binge drinking approximately once a week and consumed an average of seven drinks per binge drinking episode, resulting in 17.5 billion total binge drinks, or 467 total binge drinks per adult who reported binge drinking (*3*). CDC analyzed 2011–2017 Behavioral Risk Factor Surveillance System (BRFSS) data to assess trends in total annual binge drinks per adult who reported binge drinking in the United States overall and in the individual states. The age-adjusted^†^ total annual number of binge drinks per adult who reported binge drinking increased significantly from 472 in 2011 to 529 in 2017. Total annual binge drinks per adult who reported binge drinking also increased significantly from 2011 to 2017 among those aged 35–44 years (26.7%, from 468 to 593) and 45–64 years (23.1%, from 428 to 527). The largest percentage increases in total binge drinks per adult who reported binge drinking during this period were observed among those without a high school diploma (45.8%) and those with household incomes <$25,000 (23.9%). Strategies recommended by the Community Preventive Services Task Force^§^ for reducing excessive drinking (e.g., regulating alcohol outlet density) might reduce binge drinking and related health risks.

BRFSS is a state-based, random-digit–dialed landline and cellular telephone survey of noninstitutionalized, civilian U.S. adults aged ≥18 years that collects data during each calendar month, yielding a representative sample for the year.^¶^ Because important disparities in binge drinking behavior are not apparent based on an assessment of binge drinking prevalence alone, a new measure of binge drinking among U.S. adults was used (*3*). For each adult who reported binge drinking, the annual number of binge drinking episodes was calculated by multiplying the past 30-day frequency of binge drinking by 12. The largest number of drinks consumed by adults who reported binge drinking during any occasion in the past 30 days was used to assess binge drinking intensity. The total annual number of binge drinks was calculated as the product of the annual number of binge drinking episodes and the binge drinking intensity among adults who reported binge drinking. Total annual binge drinks per adult who reported binge drinking was then determined by dividing total binge drinks by the weighted population estimates of U.S. adults who reported binge drinking.

To assess trends in total binge drinks per adult who reported binge drinking overall, by sociodemographic characteristics, and by state, CDC analyzed 2011–2017 BRFSS data. Total BRFSS sample sizes ranged from 441,456 (2015) to 506,467 (2011). The median survey response rates declined from 49.7% in 2011 to 45.9% in 2017.** Data were weighted to each state’s adult population and to each respondent’s probability of selection. SAS (version 9.4; SAS Institute) and SAS-callable SUDAAN (version 10.0.3; RTI International) were used to calculate the mean of total binge drinks per adult who reported binge drinking, age-adjusted to the 2000 projected U.S. population. Linear and quadratic trends of the total annual binge drinks per adult who reported binge drinking were assessed by orthogonal polynomial contrast; only linear trends were consistent with the temporal distribution of the study data and were reported. Two-tailed t-tests were used to assess the statistical significance (p<0.05) of linear trends overall and among specific subgroups.

The age-adjusted prevalence of binge drinking decreased from 18.9% in 2011 to 18.0% in 2017 (Table 1). However, the overall age-adjusted total annual number of binge drinks per adult who reported binge drinking increased significantly (12.1%) from 472 in 2011 to 529 in 2017 (Figure). The total number of binge drinks per adult who reported binge drinking also significantly increased from 2011 to 2017, both for men (from 587 to 666) and women (from 256 to 290) (Table 1). During this period, the total number of binge drinks per adult who reported binge drinking also increased significantly: from 468 to 593 among those aged 35–44 years, from 428 to 527 among those aged 45–64 years, from 416 to 490 among those aged ≥65 years, and from 487 to 539 among non-Hispanic white adults. In addition, the total number of binge drinks per adult who reported binge drinking increased significantly among persons with some college education or less and across all income categories. However, from 2011 to 2017, the largest percentage increases in total number of binge drinks per adult who reported binge drinking were among those with less than a high school diploma (45.8%; from 646 to 942) and those with household incomes <$25,000 (23.9%; from 543 to 673).

**TABLE 1:** Age-adjusted* binge drinking prevalence,^†^ frequency,^§^ intensity,^¶^ and total binge drinks per adult who reported binge drinking** among adults aged ≥18 years,^††^ by selected characteristics and year — Behavioral Risk Factor Surveillance System, United States,^§§^ 2011–2017

Characteristic	Year Mean (95% CI)							Linear trend p-value
	2011	2012	2013	2014	2015	2016	2017	
	(n = 36,759,000)^¶¶^	(n = 35,765,000)^¶¶^	(n = 35,044,000)^¶¶^	(n = 33,465,000)^¶¶^	(n = 35,084,000)^¶¶^	(n = 36,617,000)^¶¶^	(n = 36,896,000)^¶¶^	
**Binge drinking prevalence %^†^**	18.9 (18.6–19.1)	17.5 (17.3–17.8)	17.2 (17.0–17.5)	16.7 (16.5–17.0)	17.1 (16.9–17.4)	17.8 (17.5–18.0)	18.0 (17.7–18.2)	<0.01
**Binge drinking frequency^§^**	4.2 (4.1–4.3)	4.3 (4.2–4.4)	4.5 (4.4–4.6)	4.4 (4.3–4.5)	4.4 (4.3–4.5)	4.6 (4.5–4.7)	4.6 (4.5–4.7)	<0.001
**Binge drinking intensity^¶^**	7.2 (7.1–7.3)	7.1 (7.0–7.2)	7.2 (7.1–7.3)	7.2 (7.1–7.3)	7.0 (6.9–7.1)	7.1 (7.0–7.1)	7.1 (7.0–7.2)	<0.01
**Total binge drinks per adult who reported binge drinking**								
Overall*	472 (455–489)	473 (456–489)	497 (478–516)	501 (481–521)	493 (473–512)	516 (497–535)	529 (505–552)	<0.001
Sex*								
Men	587 (564–611)	586 (562–610)	620 (594–647)	625 (597–653)	615 (586– 644)	641 (612–669)	666 (632–700)	<0.001
Women	256 (239–272)	261 (245–277)	267 (249–285)	272 (250–294)	267 (250– 284)	299 (280–317)	290 (266–314)	<0.001
**Age group (yrs)**								
18–24	619 (557–681)	538 (495–581)	558 (512–604)	553 (501–605)	531 (483–579)	542 (481–603)	545 (483–607)	NS
25–34	496 (461–531)	491 (449–534)	532 (486–579)	520 (473–566)	501 (452–551)	479 (448–509)	479 (442–515)	NS
35–44	468 (430–505)	492 (449–534)	494 (455–533)	513 (465–562)	491 (451–532)	531 (485–577)	593 (530–655)	<0.01
45–64	428 (406–451)	462 (438–487)	480 (450–510)	497 (466–528)	483 (452–514)	552 (517–587)	527 (488–567)	<0.001
≥65	416 (367–465)	397 (358–437)	447 (394–501)	434 (383–485)	473 (411–535)	454 (407–500)	490 (424–556)	<0.05
Race/Ethnicity*^,^***								
White	487 (468–506)	485 (468–503)	506 (486–525)	527 (503–551)	503 (482–525)	529 (509–549)	539 (513–565)	<0.001
Black	386 (339–433)	421 (365–477)	429 (373–486)	392 (338–446)	430 (360–499)	415 (367–463)	433 (377–489)	NS
Hispanic	448 (367–530)	409 (352–466)	470 (394–546)	420 (369–472)	428 (359–497)	464 (396–531)	461 (390–533)	NS
American Indian/Alaska Native	725 (474–975)	753 (528–977)	688 (486–890)	885 (467–1,302)	738 (483–994)	803 (620–987)	1,179 (729–1,629)	NS
Asian/Pacific Islander	399 (225–573)	392 (267–517)	337 (247–428)	299 (183–415)	539 (194–885)^†††^	355 (200–511)	421 (314–528)	NS
Education level*								
Less than high school diploma	646 (573–719)	682 (600–764)	685 (604–765)	717 (628–806)	786 (670–902)	766 (675–858)	942 (815–1,069)	<0.001
High school diploma	565 (530–600)	545 (515–574)	604 (565–643)	600 (561–639)	585 (546–624)	642 (597–688)	647 (594–699)	<0.01
Some college	442 (412–472)	453 (427–480)	481 (450–512)	489 (456–522)	460 (430–491)	485 (457–513)	501 (463–539)	<0.05
College graduate	327 (308–345)	334 (314–354)	329 (310–348)	335 (308–361)	334 (315–353)	340 (322–357)	317 (301–333)	NS
Annual household income*								
<$25,000	543 (504–581)	596 (549–642)	598 (549–646)	648 (589–706)	590 (538–643)	590 (545–636)	673 (596–750)	<0.05
$25,000–$49,999	512 (481–544)	482 (450–513)	518 (482–554)	540 (496–583)	528 (483–573)	608 (558–658)	569 (515–622)	<0.01
$50,000–$74,999	462 (414–511)	448 (411–484)	493 (449–538)	475 (430–521)	489 (442–536)	509 (465–553)	519 (465–573)	<0.05
≥$75,000	413 (379–447)	413 (386–439)	435 (402–467)	425 (393–457)	440 (403–477)	455 (427–483)	457 (422–493)	<0.05

**Abbreviations**: CI = confidence interval; NS = not significant.

* Age-adjusted mean of total binge drinks per adult who reported binge drinking was standardized to the projected 2000 U.S. Census population.

^†^ Binge drinking was defined as consumption of five or more drinks on an occasion for men and four or more drinks on an occasion for women, during the past 30 days.

^§^ Average number of binge-drinking episodes reported by all adults who reported binge drinking during the past 30 days.

^¶^ Average largest number of drinks consumed by adults who reported binge drinking on any occasion during the past 30 days.

** Total number of binge drinks was calculated by multiplying the frequency of binge drinking (i.e., total annual number of binge drinking episodes) by the binge drinking intensity (i.e., the largest number of drinks consumed by adults who reported binge drinking on any occasion) for each adult who reported binge drinking.

^††^ Including respondents aged 18–20 years who are under the legal drinking age.

^§§^ Respondents were from all 50 states and the District of Columbia.

^¶¶^ Weighted total population of adults who reported binge drinking.

*** Whites, blacks, American Indian/Alaska Natives, and Asian/Pacific Islanders were non-Hispanic; Hispanic persons could be of any race.

^†††^ Unreliable estimates if relative standard error >0.3 or n<50.

**FIGURE:**
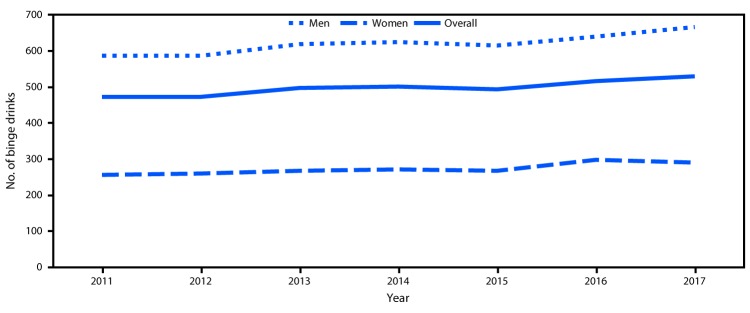
Age-adjusted* annual number of binge drinks per adult who reported binge drinking^†^ among adults aged ≥18 years,^§^ by sex — Behavioral Risk Factor Surveillance System, United States,^¶^ 2011–2017 * Age-adjusted mean of total binge drinks per adult who reported binge drinking was standardized to the projected 2000 U.S. Census population. ^†^ Total number of binge drinks was calculated by multiplying the frequency of binge drinking (i.e., total annual number of binge drinking episodes) by the binge drinking intensity (i.e., the largest number of drinks consumed by adults who reported binge drinking on any occasion) for each adult who reported binge drinking. ^§^ Including respondents aged 18–20 years who were under the legal drinking age. ^¶^ Respondents were from all 50 states and the District of Columbia.

In 2017, the total number of binge drinks per adult who reported binge drinking ranged from 320 in Massachusetts to 1,219 in Wyoming (Table 2). From 2011 to 2017, total number of binge drinks per adult who reported binge drinking increased significantly in nine states (Idaho, Indiana, Maine, Montana, New Jersey, New York, North Dakota, Ohio, and Virginia), decreased significantly in Massachusetts and West Virginia, and did not change significantly in the other 39 states and the District of Columbia.

**TABLE 2:** Age-adjusted* total number of binge drinks per adult who reported binge drinking^†^ among adults aged ≥18 years,^§^ by state — Behavioral Risk Factor Surveillance System, United States, 2011–2017

State	Year Mean (95% CI)							Linear trend p-value
	2011	2012	2013	2014	2015	2016	2017	
Alabama	520 (257–783)	530 (423–637)	414 (320–507)	457 (375–539)	570 (450–690)	481 (400–562)	451 (296–606)	NS
Alaska	535 (341–729)	466 (369–562)	640 (415–865)	702 (529–875)	649 (447–852)	405 (326–484)	683 (376–989)	NS
Arizona	412 (335–489)	405 (333–476)	729 (486–972)	499 (409–589)	547 (403–690)	522 (431–613)	492 (419–566)	NS
Arkansas	710 (332–1,088)	732 (479–985)	748 (334–1,161)	449 (357–541)	819 (552–1,086)	843 (479–1,207)	774 (512–1,036)	NS
California	417 (359–476)	372 (323–420)	445 (370–520)	470 (391–549)	400 (342–459)	430 (358–502)	470 (379–562)	NS
Colorado	390 (334–445)	409 (353–464)	450 (385–516)	403 (352–453)	426 (367–486)	430 (358–502)	434 (368–500)	NS
Connecticut	410 (306–514)	483 (357–608)	455 (333–578)	402 (324–480)	565 (412–719)	489 (329–648)	365 (287–442)	NS
Delaware	543 (378–709)	459 (372–545)	432 (339–525)	482 (351–614)	435 (319–551)	560 (391–729)	640 (301–979)	NS
District of Columbia	353 (281–425)	325 (264–387)	354 (289–419)	379 (273–484)	323 (252–394)	342 (265–418)	334 (267–401)	NS
Florida	497 (421–573)	513 (422–603)	559 (485–632)	511 (426–596)	455 (378–531)	617 (453–781)	619 (489–749)	NS
Georgia	487 (394–579)	548 (411–685)	529 (404–654)	535 (425–646)	496 (377–616)	576 (424–727)	473 (367–580)	NS
Hawaii	636 (476–796)	703 (594–812)	634 (514–755)	577 (493–661)	635 (529–741)	646 (512–781)	622 (520–724)	NS
Idaho	433 (329–538)	434 (360–509)	556 (429–682)	448 (339–558)	533 (402–663)	520 (404–636)	793 (506–1,079)	<0.05
Illinois	497 (424–571)	499 (396–602)	525 (426–623)	517 (415–620)	451 (370–532)	532 (428–637)	441 (363–519)	NS
Indiana	482 (397–566)	511 (430–592)	562 (455–669)	582 (453–711)	521 (412–631)	625 (517–733)	699 (588–810)	<0.01
Iowa	580 (481–679)	466 (398–535)	568 (471–664)	560 (433–688)	523 (435–611)	553 (468–639)	586 (499–672)	NS
Kansas	480 (420–539)	532 (444–619)	516 (463–569)	495 (422–568)	475 (423–526)	570 (470–669)	505 (429–582)	NS
Kentucky	641 (527–756)	797 (630–964)	575 (471–679)	763 (577–950)	722 (585–858)	833 (593–1,072)	699 (554–843)	NS
Louisiana	522 (422–623)	581 (431–730)	635 (413–858)	522 (343–702)	609 (475–742)	416 (329–504)	505 (402–609)	NS
Maine	518 (437–600)	489 (416–562)	508 (418–597)	567 (450–684)	510 (435–586)	595 (487–703)	762 (503–1,021)	<0.05
Maryland	450 (324–576)	391 (336–446)	468 (374–561)	374 (310–437)	477 (365–589)	442 (382–501)	477 (384–571)	NS
Massachusetts	416 (369–463)	499 (420–578)	448 (377–518)	471 (387–555)	440 (333–547)	386 (319–452)	320 (267–372)	<0.01
Michigan	567 (473–661)	478 (399–556)	468 (413–523)	602 (494–711)	609 (491–727)	582 (475–690)	531 (454–608)	NS
Minnesota	400 (352–447)	421 (366–475)	445 (385–504)	410 (352–467)	452 (408–496)	427 (378–475)	409 (365–453)	NS
Mississippi	665 (502–827)	512 (412–612)	631 (496–766)	521 (372–669)	761 (425–1,097)	622 (449–794)	640 (437–842)	NS
Missouri	535 (433–636)	479 (371–588)	614 (438–791)	592 (456–728)	653 (488–819)	603 (499–708)	493 (408–578)	NS
Montana	467 (403–530)	481 (418–544)	454 (394–514)	550 (435–665)	498 (398–598)	475 (377–572)	658 (503–813)	<0.05
Nebraska	460 (419–502)	526 (463–589)	500 (426–574)	472 (417–528)	472 (385–559)	479 (413–545)	477 (414–540)	NS
Nevada	480 (377–582)	470 (389–551)	677 (487–868)	448 (333–564)	623 (377–868)	421 (304–538)	483 (341–624)	NS
New Hampshire	530 (348–712)	586 (408–764)	399 (328–470)	458 (355–560)	414 (331–497)	479 (388–571)	506 (366–647)	NS
New Jersey	438 (330–546)	344 (287–402)	355 (311–399)	394 (335–452)	429 (234–624)	473 (352–595)	563 (436–690)	<0.05
New Mexico	442 (376–508)	512 (427–597)	480 (407–552)	580 (478–682)	440 (351–528)	512 (369–654)	558 (428–688)	NS
New York	364 (293–435)	370 (303–438)	368 (316–420)	375 (281–469)	389 (344–435)	448 (401–495)	481 (400–561)	<0.01
North Carolina	483 (384–582)	463 (397–529)	465 (374–556)	464 (351–577)	434 (356–511)	523 (376–671)	445 (253–636)	NS
North Dakota	436 (336–535)	471 (389–553)	459 (396–523)	624 (462–785)	547 (444–649)	610 (506–713)	505 (434–576)	<0.05
Ohio	474 (402–546)	541 (466–616)	488 (428–548)	606 (481–731)	608 (444–772)	633 (527–738)	764 (603–925)	<0.01
Oklahoma	604 (459–748)	583 (490–675)	616 (465–767)	539 (438–641)	555 (417–693)	563 (373–753)	490 (389–592)	NS
Oregon	455 (361–549)	457 (358–557)	508 (400–615)	406 (335–477)	400 (322–479)	383 (325–442)	425 (356–494)	NS
Pennsylvania	472 (406–537)	497 (412–582)	599 (492–707)	450 (376–525)	471 (351–590)	505 (422–589)	584 (454–715)	NS
Rhode Island	370 (290–449)	427 (346–508)	416 (338–494)	407 (325–490)	562 (271–853)	435 (331–538)	533 (378–688)	NS
South Carolina	537 (431–643)	595 (455–735)	512 (423–602)	519 (436–602)	625 (529–721)	478 (408–548)	510 (437–584)	NS
South Dakota	423 (338–507)	497 (396–597)	458 (365–551)	456 (311–602)	425 (344–505)	491 (357–625)	590 (439–742)	NS
Tennessee	421 (214–628)	428 (321–536)	319 (196–443)	505 (335–676)	529 (395–664)	534 (423–646)	497 (367–626)	NS
Texas	525 (431–620)	512 (430–594)	536 (448–623)	545 (450–640)	516 (425–608)	546 (462–630)	568 (458–679)	NS
Utah	554 (459–649)	471 (394–549)	576 (457–694)	667 (530–803)	457 (389–525)	630 (503–757)	549 (442–656)	NS
Vermont	473 (395–551)	454 (357–551)	472 (372–572)	627 (335–919)	488 (381–595)	685 (540–831)	490 (395–585)	NS
Virginia	409 (343–476)	441 (364–517)	440 (367–513)	538 (431–645)	523 (409–637)	562 (450–674)	531 (439–624)	<0.01
Washington	374 (319–429)	482 (349–615)	444 (382–506)	441 (362–519)	384 (321–447)	427 (363–491)	428 (376–480)	NS
West Virginia	792 (575–1,009)	761 (573–949)	799 (639–959)	886 (602–1,171)	517 (419–614)	766 (574–959)	565 (450–679)	<0.05
Wisconsin	511 (392–631)	514 (421–607)	452 (393–511)	490 (385–594)	493 (414–572)	460 (390–529)	478 (378–578)	NS
Wyoming	617 (448–787)	547 (371–723)	686 (488–884)	541 (357–725)	431 (336–526)	513 (355–672)	1,219 (586–1,852)	NS

**Abbreviations:** CI = confidence interval; NS = not significant.

* Age-adjusted mean of total binge drinks per adult who reported binge drinking was standardized to the projected 2000 U.S. Census population.

^†^ Total number of binge drinks was calculated by multiplying the frequency of binge drinking (i.e., total annual number of binge drinking episodes) by the binge drinking intensity (i.e., the largest number of drinks consumed by adults who reported binge drinking on any occasion) for each adult who reported binge drinking.

^§^ Including respondents aged 18–20 years who are under the legal drinking age.

## Discussion

The total annual number of binge drinks consumed per U.S. adult who reported binge drinking increased significantly by 12% from 2011 to 2017, including among non-Hispanic white adults and those aged ≥35 years. These increases are consistent with other recent evidence of an approximately 30% increase in high-risk drinking,^††^ including binge-level alcohol consumption, particularly among middle-aged and older adults (*4*). Because binge drinking contributes a substantial proportion of all alcohol consumption in the United States, these increases also are consistent with an increase in per capita alcohol consumption (derived from sales and shipment data) in the United States,^§§^ from 2.29 gallons in 2011 to 2.34 gallons in 2017.

The finding that the total number of binge drinks consumed per U.S. adult who reported binge drinking increased significantly among those with lower education and income levels is also consistent with a recent study that found the majority of persons reporting prescription opioid misuse also are adults who reported binge drinking, and that prescription opioid misuse tends to be most common among persons with lower household incomes (*5*). Socioeconomic disparities in the total number of binge drinks per adult who reported binge drinking also might have contributed to the lower life expectancies reported among persons with lower socioeconomic status in the United States (*6*).

The total annual number of binge drinks per adult who reported binge drinking did not change significantly in most states from 2011 to 2017, although it did increase significantly in nine states. At the state or local levels, examining the total number of binge drinks consumed by adults who reported binge drinking is a relatively new way to assess binge drinking and related harms. However, by combining public health surveillance data on the prevalence, frequency, and intensity of binge drinking, this measure provides a more complete and sensitive indicator of this health risk and facilitates assessment of sociodemographic and geographic disparities in binge drinking. This measure also might be useful for assessing health risks related to binge drinking (e.g., opioid misuse) (*5*), and for planning and evaluating effective strategies for preventing binge drinking at the state and local levels.

The findings in this report are subject to at least four limitations. First, BRFSS data are self-reported, and the BRFSS substantially underestimates alcohol consumption in the United States relative to alcohol sales data (*7*). Second, the BRFSS measure of the largest number of drinks among adults who reported binge drinking might have resulted in higher estimates of binge drinking intensity than would other survey methods, such as when collecting information on the most recent binge drinking episode for adults who reported binge drinking, including the number of drinks consumed by beverage type (*8*). However, because the underreporting of alcohol consumption tends to be greater among binge drinkers than among non-binge drinkers and tends to increase with binge drinking intensity (*9*), the prevalence, frequency, and intensity of binge drinking are likely to have been substantially underestimated in this study. Third, similar to other telephone surveys, BRFSS response rates have been declining, which could affect the representativeness of the survey responses. However, BRFSS response rates did not change substantially during the study period, and were, therefore, unlikely to have affected trends. Finally, BRFSS does not survey institutionalized adults, which limits the generalizability of the findings to noninstitutionalized persons.

Reducing binge drinking is essential to reducing excessive drinking at the population level. These findings highlight the need to reduce the total number of binge drinks per adult who reported binge drinking by reducing the prevalence, frequency, and intensity of binge drinking. Moreover, monitoring binge drinking prevalence alone, the most commonly used measure of binge drinking, portrays an incomplete picture of the problem of binge drinking, and might mask important sociodemographic and socioeconomic disparities in binge drinking behavior. Binge drinking is also strongly affected by the social context within which persons make their drinking decisions. For example, persons living in states with more restrictive alcohol policies are also less likely to binge drink and experience alcohol-attributable harms, including motor vehicle crash deaths, alcoholic liver cirrhosis, and alcohol-involved homicides and suicides than are persons living in states with less restrictive alcohol policies (*10*). Evidence-based prevention strategies to decrease excessive drinking that the Community Preventive Services Task Force recommends include increasing alcohol taxes, regulating the number and concentration of alcohol outlets in communities, and enforcing minimum legal drinking age laws.

**Summary**

**What is already known about this topic?**

In 2015, 37 million (17.1%) U.S. adults reported binge drinking approximately once a week and consumed an average of seven drinks per binge drinking episode, resulting in approximately 450 total binge drinks per adult who reported binge drinking annually.

**What is added by this report?**

From 2011 to 2017, the total number of binge drinks consumed annually by U.S. adults who reported binge drinking increased significantly, from 472 to 529. Significant increases were observed among adults who reported binge drinking of both sexes, those aged ≥35 years, and those with lower educational levels and household incomes.

**What are the implications for public health practice?**

Application of population-level evidence-based prevention strategies (e.g., regulating alcohol outlet density) could reduce binge drinking and related harms.

## Footnotes

* Excessive alcohol consumption includes binge drinking (i.e., five or more drinks on an occasion for men and four or more drinks on an occasion for women), heavy weekly alcohol consumption (i.e., 15 or more drinks per week for men; eight or more drinks per week for women), and any drinking by pregnant women or those aged <21 years. https://www.cdc.gov/alcohol/fact-sheets/alcohol-use.htm.

† https://www.cdc.gov/nchs/data/statnt/statnt20.pdf.

§ https://www.thecommunityguide.org/topic/excessive-alcohol-consumption.

¶ https://www.cdc.gov/brfss/annual_data/2017/pdf/overview-2017-508.pdf.

** Response rates for BRFSS are calculated using standards set by the American Association for Public Opinion Research response rate formula 4 (https://www.aapor.org/AAPOR_Main/media/publications/Standard-Definitions20169theditionfinal.pdf). The response rate is the number of respondents who completed the survey as a proportion of all eligible and likely eligible persons.

†† High-risk drinking was defined as drinking four or more standard drinks on any day for women or five or more standard drinks on any day (not necessarily during one sitting) for men.

§§ https://pubs.niaaa.nih.gov/publications/surveillance113/CONS17.htm.
